# Diabetes Eye Disease Sufferers and Non-Sufferers Are Differentiated by Sleep Hours, Physical Activity, Diet, and Demographic Variables: A CRT Analysis

**DOI:** 10.3390/healthcare12232345

**Published:** 2024-11-23

**Authors:** Damián Pereira-Payo, Ángel Denche-Zamorano, María Mendoza-Muñoz, Raquel Pastor-Cisneros

**Affiliations:** 1 Health, Economy, Motricity and Education (HEME) Research Group, Faculty of Sport Sciences, University of Extremadura, 10003 Cáceres, Spain; dpereirapayo@unex.es; 2 Promoting a Healthy Society Research Group (PHeSO), Faculty of Sport Sciences, University of Extremadura, 10003 Cáceres, Spain; raquelpc@unex.es; 3 Physical and Health Literacy and Health-Related Quality of Life (PHYQoL), Faculty of Sport Science, University of Extremadura, 10003 Cáceres, Spain

**Keywords:** retinopathy, exercise, sedentarism, nutrition, CART

## Abstract

Introduction: Diabetic eye disease is the most common microvascular complication of diabetes mellitus. This complication has some direct impact on an individual’s well-being and health. Some lifestyle habits have been associated with the incidence of these co-morbidities. Objective: To classify the diabetic population into sufferers or non-sufferers of diabetes eye disease according to lifestyle and demographic variables, and to identify which of these variables are significant for this classification. Methods: The present cross-sectional study based on the NHANES 2011–2020 used the Classification and Regression Tree (CRT) analysis for classifying the diabetic population into sufferers and non-sufferers of diabetes eye disease. The odds ratio (OR) and relative risks (RR) of suffering this diabetes complication of the subgroups formed by the model were studied. The final sample formed 2657 individuals (1537 males and 1120 females). Results: A 79.4% accuracy was found for the CRT model. The independent variables of sleep hours (100.0%), physical activity (PA) group (92.8%), gender (76.2%), age (46.4%), education level (38.4%), sedentary time (38.1%), and diet (10.0%) were found to be significant for the classification of cases. The variable high alcohol consumption was not found significant. The analysis of the OR and RR of the subgroups formed by the model evidenced greater odds of suffering diabetes eye disease for diabetes sufferers from the inactive and walk/bicycle PA group compared to those from the Low, Moderate, and High PA groups (OR: 1.48 and RR: 1.36), for males compared to females (OR: 1.77 and RR: 1.61), for those sleeping less than 6 h or more than 9 compared to those who sleep between 6 and 8 h (OR: 1.61 and RR: 1.43), and for diabetes sufferers aged over 62 compared to younger ones (OR: 1.53 and RR: 1.40). Conclusions: sleep hours, PA group, gender, age, education level, sedentary time, and diet are significant variables for classifying the diabetic population into sufferers and non-sufferers of diabetes eye disease. Additionally, being in the inactive or walk/bicycle PA group, being a male, sleeping less than 6 or more than 9 h, and being aged over 62 were identified as risk factors for suffering this diabetes complication.

## 1. Introduction

Diabetes mellitus (DM) is a group of non-communicable metabolic diseases, whose sufferers are characterized as having elevated blood sugar levels during prolonged periods of time [1]. People affected by this disease are not able to endogenously manage their blood glucose levels [1], making blood glucose homeostasis difficult. Depending on the type of diabetes suffered, blood glucose deregulation may occur due to the inability to produce insulin in type 1 DM [2,3], or due to insulin resistance in type 2 DM [3].

Worldwide, the prevalence of diabetes is 422 million people according to the World Health Organization [4]. In the US, 38.4 million individuals suffer this pathology [5]. DM entails high economic costs adding up to a total of USD 327 billion according to the American Diabetes Association’s estimations [6].

When DM is untreated or poorly managed, it translates into prolonged periods of hyperglycemia, which may cause a series of short-term symptoms, such as thirst, enhanced urination frequency, blurred vision, tiredness, and unintentional weight loss [4]. However, it can also contribute to the development of a series of co-morbidities over time, such as neuropathy, nephropathy, cardiovascular disease, and retinopathy [7].

Diabetic eye disease is the most common microvascular complication of the disease, with one third of all sufferers worldwide being affected by it [8], and 26.43% of them in the US [9]. It should also be noted that, although diabetic retinopathy is the leading cause of sight loss in people with diabetes, other retinal and non-retinal pathologies can also affect eyesight in this population [10]. Those who suffer this disease complication have been found to experience reduced well-being and health-related quality of life, poorer mental health, and ultimately difficulties in basic everyday tasks, such as walking, driving, reading, and even working [11,12,13]. In this sense, vision affected by diabetes entails an average cost of USD 16,838 per person per year in the United States [14].

Some lifestyle habits have been associated with a better management of diabetes mellitus disease, and a reduced development of its co-morbidities, such as affected eyesight. Sleep habits [15], diet quality [16], alcohol consumption [17], and physical activity involvement [16,18] have been shown to have a major influence on the onset of diabetes symptoms, specifically vision problems.

In terms of the lifestyle of diabetes patients, sleep habits have been shown to have an important effect on the disease symptomatology [19]. A reduction in the hours of sleep has been shown to be a factor in the appearance of several symptoms of this pathology, as well as poor metabolic control in both DM types [20]. Short sleep duration, obstructive sleep apnea, shift work, and insomnia are also associated with an increased risk of type 2 diabetes and may predict worse outcomes in people with existing diabetes [21]. Poor sleep habits, such as short sleep or excessive daytime sleepiness, can lead to health contraindications in this population, such as increased BMI or intensive anti-diabetic treatment [19].

Eating behaviours are of crucial importance in the management of diabetes, and in the appearance of its symptoms [22]. The prevention of diabetic retinopathy must include the adequate control of blood glucose, lipid levels, and blood pressure, which can be achieved by promoting changes in dietary habits [23]. It has been found that the occurrence of vision problems in people with diabetes is associated with the quality of the diet [24]. Previous studies suggest that an adherence to the Mediterranean diet and a high consumption of fruit, vegetables, and fish may protect against the development of diabetic retinopathy [25,26].

Regarding alcohol consumption, its chronic consumption has been associated with an increased incidence of diabetic retinopathy [27] and a reduced risk of progression and development of this disease [28]. A previous review study has reported a significant association between chronic alcohol consumption and an increased incidence of eye diseases such as an increased risk of cataracts, age-related macular degeneration, diabetic retinopathy, different types of optic neuropathy, impaired visual quality, retinal vascular disease, and ocular surface disease [17].

An active lifestyle has proven to be one of the best allies for good diabetes control [29]. Greater PA involvement was found to be associated with a reduced incidence of vision problems in the diabetic population [18]. A previous study has shown that higher levels of physical activity are associated with a lower risk of developing vision problems such as diabetic retinopathy [30]. It has also been shown that physical activity levels are lower in people with poorer vision, and that moderate-intensity physical activity may have a small protective effect in preventing certain eye diseases [18]. On the other hand, a sedentary lifestyle leads to a worse management of the pathology [31], and is associated with a greater risk of developing disease comorbidities, and specially diabetes-affected eyes [32].

Demographic characteristics have been found to have importance in terms of diabetes symptomatology. In terms of gender, males have a higher prevalence of diabetes than females. Regarding diabetes co-morbidities, it has been found that men have a higher prevalence of vision problems caused by diabetes than females [33]. For its part, educational level has been shown to be associated with diabetes symptomatology, and with diabetes retinopathy in particular [34].

In light of the above, the hypothesis of this study was that diet, physical activity, sleep hours, sedentary time, and the demographic variables of education level and gender are significant variables to classify people with diabetes into those with diabetes eye disease or not.

Decision trees allow the interactions between risk factors to be understood and high-risk subgroups to be identified for the complication studied [35]. Previous investigations have identified some individual risk factors for developing diabetic eye disease in diabetes sufferers, some of them being: sleep habits [15], diet quality [16], alcohol consumption [17], physical activity involvement [16,18], gender [33], and educational level [34]. In contrast, we have not found any article that has analyzed the interactions of all these risk factors through classification and regression trees. Therefore, the objectives of our study were: to analyze the lifestyle habits that are predictors of diabetic eye disease in diabetes sufferers, to identify groups at high risk of this diabetes complication, and to calculate the odds ratios and relative risks of developing diabetes eye disease according to these.

## 2. Materials and Methods

### 2.1. Design

This cross-sectional study is based on data from the NHANES (National Health and Nutrition Examination Survey), in its editions from 2011 to 2020. The NHANES is a bi-annual program that aims to generate national representative data of the noninstitutionalized US population [36]. It is conducted by the Centers for Disease Control and Prevention’s (CDC) National Center for Health Statistics (NCHS). The selected individuals who agree to participate undergo a medical examination and a survey which is conducted and supervised by NHANES professionals [36]. NHANES staff are encompass dietary professionals, health interviewers, physicians, and medical and health technicians [36].

### 2.2. Participants

Participants from the NHANES 2011–2020 were selected based on a stratified, clustered four-stage sampling system. All information regarding sampling, the treatment of missing data, and all efforts made to address the potential sources of bias is described in [37,38,39].

The total number of participants in the NHANES from 2011 to 2020 was 45,462 people (males = 22,472 and females = 22,990). The selection criteria for this research were: suffering diabetes, being 20 years of age or older, and having valid data for the following variables: diabetic eye disease (item DIQ080), gender (item RIAGENDR), diet (item DBQ700), high alcohol consumption (item ALQ151), sleep hours (items SLD012 and SLD013, or SLD010H in NHANES 2011–2012 and 2013–2014), PA group (PAQ610, PAD615, PAQ625, PAD630, PAQ640, PAD645, PAQ655, PAD660, PAQ670, and PAD675), and sedentary time (item PAD680).

For the final sample, 42,805 participants were excluded, 18,982 for being younger than 20, and the other 22,590 participants were excluded for not having diabetes, 37 for not having valid data for diabetic eye disease, 946 for not having valid data for high alcohol consumption, 31 for not having valid data for sleep hours, and 19 for not having valid data for PA group and sedentary time. Thus, the final sample was formed of 2657 individuals (1537 males and 1120 females).

### 2.3. Variables

- Age: in years at the moment of screening. Corresponded to the item RIDAGEYR of the NHANES.
- Gender: corresponded to the item RIAGENDR of the NHANES. The possible answers were: “male” or “female”. “Male” was coded as “0”, while “female” as “1”.
- Education level: participants were asked what the highest level of education was that the participant had completed (corresponded to item DMDEDUC2). The answers could be one of the following: (1) “less than 9th grade”, (2) “9–12th grade without diploma”, (3) “High school graduate/GED”, (4) “Some college or AA degree”, and (5) “College graduate or above”. This variable was coded as follows: “less than 9th grade” = 0, “9–12th grade without diploma” = 1, “High school graduate/GED” = 2, “Some college or AA degree” = 3, and “College graduate or above” = 4.
- Diabetic eye disease: Participants were asked: “Has a doctor ever told you that diabetes has affected your eyes or that you had retinopathy?”. The possible answers were: “yes” and “no”. This corresponded to the item DIQ080. “No” was coded as “0” and “Yes” as “1”.
- Diet: Participants were asked: “how healthy is your diet?”. The possible answers were: “poor”, “fair”, “good”, “very good”, and “excellent”. This variable corresponded to the item DBQ700 of the NHANES. This variable was coded as follows: “excellent” = 0, “very good” = 1, “good” = 2, “fair” = 3, and “poor” = 4.
- High alcohol consumption: Participants were asked: “Was there ever a time or times in your life when you drank four/five or more drinks of any kind of alcoholic beverage almost every day?”. The possible answers were: “yes” and “no”. This corresponded to item ALQ151. “No” was coded as “0”, and “Yes” as “1”.
- Sleep hours: This variable was constructed with the items SL012 (sleep hours on weekdays) and SLD013 (sleep hours on weekends). SL012 was multiplied by five and SLD013 was multiplied by two; the sum of these two numbers was divided by seven, providing the average number of hours slept in the week. This variable was coded as follows: “Less than 5” h = 0, “5” h = 1, “6” h = 2, “7” h = 3, “8” h= 4, “9” h = 5, and “10 or more” h = 6.
- PA group: This variable corresponded to the Generalized Physical Activity Questionnaire [40]. In this questionnaire, participants are asked about their participation in PA of vigorous intensity (which corresponded to the items: PAQ605, PAQ610, PAD615, PAQ650, PAQ655, and PAD660), PA of moderate intensity (which corresponded to the items: PAQ620, PAQ625, PAD630, PAQ665, PAQ670, and PAD675), and walking or cycling (which corresponded to the items: PAQ635, PAQ640, and PAD645). From the answers to these questions, the metabolic equivalents (METs) were calculated, attributing 8 METs to the vigorous activities, 4 METs to the moderate activities, and 3.3 METs to the activities of walking and cycling [41,42].

According to their answers and their total METs, participants were classified into one of the following groups:

- High PA: for the participants that performed three or more days of vigorous PA reaching over 1500 METs, or accumulated seven or more days of any combination of PA types reaching 3000 METs [42].
- Moderate PA: Participants were included in this group if they did not qualify for being selected for the High PA group and met one of the following criteria: (1) three or more days of vigorous PA for 20 min a day; (2) five or more days of moderate PA, and/or 30 min a day of walking or cycling; (3) reaching 600 METs or more, with five or more days of any kind of PA [42].
- Low PA: If they did not meet the criteria for being classified as High or Moderate PA, but they performed greater PA than the walking/cycling group (only walking or cycling as a means of transportation), then they were assigned to this group.

Participants that do not meet the criteria for the groups High PA and Moderate PA, but their declared PA was higher than that of participants from the Walking and Inactives groups:

- Walking/Cycling: participants that could not be classified as “high”, “moderate”, or “low” PA, but performed one or more days of walking/cycling as a way of transportation, but did not perform any other type of PA.
- Inactives: those participants that did not perform PA of any type.

The GPAQ has been shown to be valid and reliable [40].

This variable was coded as follows: “Inactives” = 1, “Walking/Cycling ” = 1, “Low PA” = 2, “Moderate PA” = 3, “High PA” = 4.

Sedentary time: Participants were asked “how much time do you usually spend sitting on a typical day?” (minutes). This question was also extracted from the GPAQ [40], and corresponded to item PAD680.

### 2.4. Statistical Analysis

The distributions followed by the data of the interest variables were analysed using graphs and a normality test (Kolmogorov–Smirnov test). The Kolmogorov–Smirnov test and the histogram representation did not show sufficient evidence to assume that data followed a normal distribution (*p* < 0.001). Thus, non-parametric tests were used for the following analysis.

Categoric variables (diet, educational level, high alcohol consumption, sleep hours, and PA group) were presented in absolute and relative frequencies, while numeric variables (age and sedentary time) were presented in median and interquartile ranges, and, as complementary data, mean and standard deviation were also provided. The Chi-squared test was used to study associations among the categoric variables and gender, while the post-hoc pairwise z-test for independent proportions was used to study differences among males and females. Differences among genders in the numeric variables were studied through the Mann–Whitney U test.

A classification and regression tree analysis (CRT) was used to classify diabetes sufferers in those whose eyes are affected by diabetes or not, according to demographic and lifestyle variables. The CRT method searches for the best split (the independent variable that causes a greater improvement) every time, dividing a parent node into two child nodes. The model keeps dividing until the stopping criteria are triggered [43,44].

The independent variables included in the model were: sleep hours, PA group, gender, age, education level, sedentary time, and diet. The model provided a classification of these variables according to their importance in the model.

The CRT model was set with these specifications:

The Gini impurity measure was used, and the impurity measure was not allowed to be below 0.001.

Parent nodes were set to have a minimum of 200 participants, while 100 was the minimum for child nodes. When this cannot be met, the model stopped its division.

Missing data were set to be excluded, but no participant was excluded from the model for this reason, since not having missing data in the variables of interest was among the inclusion criteria.

The model accuracy was tested through a ten-fold cross-validation analysis [45].

In order to compare the results of the CRT model, a binary logistic regression model was performed via the backward stepwise approach. The dependent variable was diabetic eye disease, and the independent variables were: age, gender, diet, education level, high alcohol consumption, sleep hours, PA group, and sedentary time.

The significance level was set at *p* < 0.05.

The statistical procedures were performed with the software SPPS in its 26 version (IBM SPSS, Chicago, IL, USA), while the level of significance of 0.05 was assumed.

## 3. Results

Participants had a median age of 63 years (IQR = 60), and significant differences in age were found among genders (*p* < 0.001). Dependence relationships were found between gender and diet, education level, high alcohol consumption, and the PA group (*p* < 0.001). Regarding diet, males and females presented differences in proportions regarding the way they perceived their diet, with more females perceiving their diet as “poor” (11.4% vs. 5.4%, *p* < 0.05) and more males defining theirs as “very good” (18.5% vs. 14.7%, *p* < 0.05) or “excellent” (8.3% vs. 3.4%, *p* < 0.05). Regarding high alcohol consumption, a significantly greater portion of males drank or had drunk 4, 5 or more drinks daily (27.0% vs. 10.9, *p* < 0.05). In terms of educational level, the group with the highest proportion of participants was the college or AA degree; regarding gender differences, a significantly higher percentage of females than males were in this group (34.0% vs. 29.3%, *p* < 0.05). Similarly, gender proportion differences were found for the remaining education levels (*p* < 0.05), except for the high school graduate/GED level where no gender differences were found. Among the remaining education levels, females were significantly more prevalent in the 9–11–12th grade with no diploma (16.5% vs. 13.3%, *p* < 0.05), while males had the highest prevalence in the lower than 9th grade (14.3% vs. 9.3%, *p* < 0.05) and college graduate or above education levels (20.3% vs. 15.4%, *p* < 0.05). In the PA group, significant proportion differences were identified among genders; in the Inactives group, females were more prevalent (42.5% vs. 32.8%, *p* < 0.05), while more males belonged to the High PA group (21.5% vs. 13.3%, *p* < 0.05). In contrast, no associations were found between hours of sleep and gender (*p* = 0.455). In terms of hours of sleep there were no differences among genders. Finally, regarding the minutes expended on sedentary activities there were no significant differences among genders (*p* = 0.362) (Table 1).

**Figure 1:**
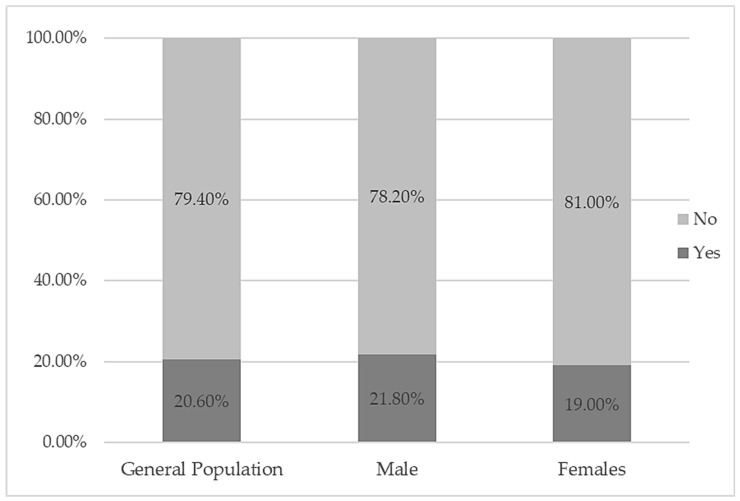
Incidence of diabetic eye disease in patients with diabetes by gender.

No associations were found between diabetes eye disease and gender (*p* = 0.081). Among people with diabetes, the prevalence of diabetes eye disease was 20.60%, with no significant differences among males and females according to the pairwise z-test for independent proportions (Figure 1).

**Figure 2:**
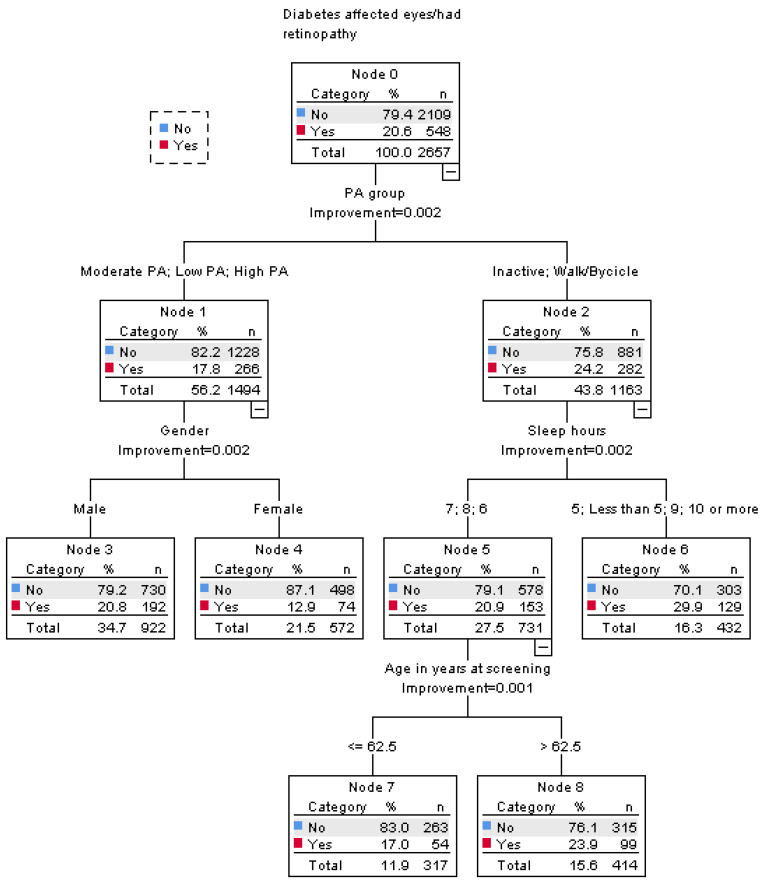
This figure shows the categories for the dependent variable (diabetes eye disease), and the eight nodes defined by the model.

The tree generated by the CRT model was formed in the following way (Figure 2):

Root node: this was split by PA group, and a lower incidence of suffering from diabetic eye disease was observed in participants from the Low, Moderate, and High PA groups (node 2; no = 82.2% and yes = 17.8%), while a higher prevalence of diabetic eye disease was seen in participants from the Inactives and Walking/Cycling PA groups (node 3; no = 75.8% and yes = 24.2%).

Branch beneath node 1: Participants from the Low, Moderate, and High PA groups were split into two sub-groups based on gender. Males (no = 79.2% and yes = 20.8%) were shown to have a greater prevalence of diabetic eye disease than females (no = 87.1 and yes = 12.9%). Females from the Low, Moderate, and High PA groups were the subgroup with the lowest prevalence of diabetic eye disease in the model.

Branch beneath node 2: Participants from the Inactives and Walking/Cycling PA groups were split by hours of sleep. The first sub-group was formed of those who slept 6, 7, or 8 h (node 4; no = 79.1% and yes = 20.9%) and had a reduced incidence of diabetic eye disease. The second sub-group, which had an increased prevalence of this diabetes complication, was formed by those with fewer than 5, 5, 9, or 10 or more hours of sleep (node 6; no = 70.1% and yes = 29.9%)—this subgroup had the highest incidence of diabetic eye in the entire model.

Finally, in Node 4, age was the splitting variable, and those aged over 62.5 years had higher prevalence of being affected by diabetic eye disease (Node 7; no = 83.0% and yes = 17.0%), while those aged 62.5 or younger had a lower prevalence (Node 8; no = 76.1% and yes = 23.9%).

The classification and regression tree model correctly classified the 79.4% of cases, as was shown by the cross-validation analysis. The variables of sleep hours (100.0%), PA group (92.8%), gender (76.2%), age (46.4%), education level (38.4%), sedentary time (38.1%), and diet (10.0%), among all variables included in the model, were identified as significant for classifying diabetes patients into sufferers or non-sufferers of diabetes eye disease (Table 2). On the other hand, the variable of high alcohol consumption was found not significant for the classification of cases.

Table 3 shows the odds ratio and relative risks of comparing the sub-groups formed by the model. Under the Root Node, participants from the Inactives and Walking/Cycling PA groups were found to have a greater odds ratio and relative risk of diabetic eye disease than those from the Low, Moderate, and High PA groups (OR: 1.48 and RR: 1.36). In the branch beneath Node 1, among participants from the Low, Moderate, and High PA activity groups, males had an increased odds ratio and relative risk of having diabetic eye disease in comparison to females (OR: 1.77 and RR: 1.61). The same occurred under Node 2 for the participants from the Inactives and Walking/Cycling PA groups who slept fewer than 5, 5, 9, or 10 h or more compared to those who slept 6, 7, or 8 h (OR: 1.61 and RR: 1.43). Finally, beneath Node 5, among participants from the Inactives and Walking/Cycling PA groups who slept 6, 7, or 8 h, those older than 62.5 years had a greater odds ratio and relative risk of suffering from diabetic eye disease than participants of 62.5 years of age or younger (OR: 1.53 and RR: 1.40).

Additionally, the binary logistic regression model identified the variables of sleep hours and PA Group as significant variables in the model (Table 4). Increased odds of suffering diabetic eye disease were seen for participants who slept fewer than five hours and for those who were in the Inactives or Walking/Cycling PA groups. This is in line with the results of the CRT model, where sleep hours and PA group were the two most important variables in the model. Other variables such as gender, age, education level, sedentary time, and diet were not part of the model, even though they were in the CRT.

## 4. Discussion

The aim of the present research was to identify which lifestyle habits and demographic variables are significant for dividing the diabetic population into those who sufferer diabetes eye disease and those who do not. The results of the present research identified the variables of sleep hours, PA group, gender, age, education level, sedentary time, and diet as significant variables in the CRT model, while in the binary logistic regression model the variables of sleep hours and PA Group were the only significant variables. Additionally, the OR and RR of having eyes affected by diabetes of the sub-groups of each branch of the CRT model were calculated. Greater odds of this problem were found in those diabetes sufferers from the Inactives and Walking/Cycling PA groups, in males, in those who slept fewer than 6 h or 9 or more hours, and in diabetes sufferers aged over 62.

The number of hours slept was the variable with the highest importance in the CRT model, and it was also significant in the binary logistic regression model. In fact, it has been found that sleep quality and quantity is a significant factor in the incidence of eye problems among people with diabetes [46]. In this manuscript, the CRT model showed significantly increased odds of having the eyesight affected for sufferers of the disease whose physical activity group was Inactives or Walking/Cycling and who slept fewer than 6 h or 9 or above. On the other hand, the binary logistic regression model showed that those who slept fewer than 5 h had twice the odds of suffering this diabetes complication than the sufferers of this disease who slept 7 h. Other researchers have found a U-form association among sleep hours and the onset of diabetic retinopathy, showing that sleep restriction but also excessive sleep hours are associated with an increased incidence of this comorbidity [15,46]. Also, short sleep duration is a significant risk factor for impaired metabolic regulation in both T1D and T2D [20], which may contribute to diabetic retinopathy progression. This may be especially relevant given that there is substantial evidence supporting the view that sleep disorders are highly prevalent in people with diabetes [47]. Furthermore, a recent review has suggested that sleep disorders are associated with poorer health outcomes in people with diabetes [47].

The second variable of importance according to the ranking of the CRT model was PA group, which was also found to be a significant variable in the binary logistic regression model. PA is known to have a crucial role in the development of diabetes symptomatology. Having a physically active lifestyle is key for the diabetic population for a better management of the disease, and for better glycaemic control [48,49]. A higher physical activity participation has been associated with a reduction in the time patients spend in hyperglycemic states and an increase in the time spent within the normal blood glucose range [50]. These findings suggest that the incorporation of physical activity interventions in the comprehensive management of diabetes could have a positive impact on patients’ glycemic control, which is fundamental for preventing complications of the disease such as diabetic retinopathy [50]. Precisely, regarding effects on eyes, a greater PA involvement has been shown to be significantly associated with a reduced prevalence of diabetes retinopathy [51]. Other benefits of physical activity include improved glycaemia, increased insulin sensitivity, and a maintenance of endothelial function [52]. So, a higher physical activity level could be associated with a lower risk of diabetic retinopathy progression [51]. Specifically, the risk of diabetic retinopathy progression could be reduced by 40% if physical activity is performed for no less than 30 min, five days a week [53].

The results of this research showed that diabetes sufferers from the Inactives and Walking/Cycling PA groups had greater odds of having their eyes affected by the disease, in comparison to those from the Low, Moderate, and High PA groups. Greater odds of diabetes symptomatology have been shown for physically inactive people [54]. Walking is a form of PA that is often suggested for people with diabetes [55], but official recommendations tend to support physical activity with greater volume and intensity for diabetes sufferers [56]. A lower prevalence of diabetic macular oedema has been observed in the diabetic population with moderate physical activity [57]. Additionally, being in the high PA group was associated with a lower incidence of diabetic retinopathy [58], which is consistent with the results of our study.

The other PA variable that was significant in the model was sedentary time. Associations among time spend in sedentary activities and the prevalence of retinopathy have been reported in previous studies [54,59]. Prolonged daily sedentary time is strongly associated with sight-threatening diabetic retinopathy [59]. Consistent with this, increased physical activity is associated with a lower risk of diabetic retinopathy [30], as well as less severe diabetic retinopathy [55]. It is worth noting that the relationship between PA and vision problems in the population with diabetes may be two-fold [18]. On the one hand, greater PA prevents the onset of vision problems, but also, vision problems may limit the PA involvement of those who suffer from them [18].

The last variable related to lifestyle habits that was significant in the CRT model was the perceived quality of diet. Although making a significant contribution to the model, diet as well as sedentary time did not appear in the tree spreading any node, and neither was identified as a significant variable in the binary logistic regression model. The dependence relationship among diet and diabetes symptomatology has been studied [60]. Regarding affected vision, an adequate diet has been shown to significantly reduce the risk of the onset of diabetic retinopathy [16,61]. In addition, patients with diabetic retinopathy have been shown to be more often deficient in essential vitamins and minerals, which increases homocysteine levels and oxidative stress, leading to an increased risk of microvascular damage [61,62]. Therefore, an adequate intake of fish could inhibit the development of retinopathy, while the consumption of fruit and vegetables could reduce the risk of damage to retinal nerve cells [63,64]. Obesity could be a relevant diet-related factor, as a 2018 meta-analysis found that obesity increased the incidence of diabetic retinopathy in patients with DM2 [65]. However, obesity was not found to be related to diabetic retinopathy progression [16]. Thus, in relation to diet, the evidence suggests that individuals who improved their dietary habits and also increased their physical activity experienced a decrease in the incidence of diabetes and its vascular complications [16].

Although some investigations have found associations between alcohol consumption and the incidence of diabetes eye disease [17,28], the variable of high alcohol consumption was not found to be significant in the CRT model, nor in the binary logistic regression model. In line with this, no significant associations among alcohol intake and the incidence of diabetes retinopathy were found in a meta-analysis including 15 studies [66].

For the demographic variables, it was found that both gender and age were significant variables in the CRT model, but not in the regression model. Gender was ranked as the third variable in importance, and male sufferers of diabetes were shown to have increased odds of having their eyes affected, in comparison to females. The relationship between sex and the incidence of ocular retinopathy, or having eyes affected by diabetes, has been studied, with a higher odds ratio found in male diabetes sufferers than in female diabetics [67,68]. The influence of sex hormones is also important in diabetic patients, especially with regard to eye diseases, with greater complications for men [69,70].

For its part, age followed gender in importance in the CRT model. Sufferers aged over 62 years with an Inactives or Walking/Cycling PA, and who sleep six to eight hours a day, were seen to have eyes that were more greatly affected. Other studies confirm our findings that older people with diabetes tend to have a higher incidence of diabetic symptoms and therefore a higher incidence of vision and retinal problems [71,72]. At the same time, the duration of diabetes is considered to be a very important risk factor for the development of diabetic retinopathy [73]. This may be the main reason why older age is associated with a higher incidence of vision problems, as older age is likely to mean that diabetes has been developing for longer than in a younger person.

### 4.1. Practical Implications

In the present research, CRT allows us to understand the phenomenon of the incidence of diabetes eye disease according to lifestyle and the demographic data of the diabetic population. Thus, one of the biggest potentials of this CRT model is that it allows us to identify potential changes in the lifestyle variables of the individuals with diabetes in order to reduce their risk of suffering this comorbidity.

In this way, the model showed that individuals who are physically inactive, or for whom walking or cycling is the only means of PA, would experience a reduction of their risk of having their eyes affected by the disease if they increased their PA participation to reach a Low, Moderate, or High PA. By achieving this, the odds of suffering diabetic eye disease would drop from 24.2% (in node 2) to 17.8 (in node 1).

Regarding sleep hours, which was identified as another modifiable risk factor, individuals affected by diabetes who were part of the Inactives or Walking/Cycling PA group, and who slept fewer than 6 h or 9 or more, would benefit from adjusting their sleep time to the range of 6 to 8 h, which, according to the present model, would reduce their odds of suffering diabetes eye disease from 29.9% (node 6) to 20.9% (node 5).

### 4.2. Limitations

The main limitation of this work is that the PA performed was not directly measured; instead, it was estimated through the GPAQ, and the same occurred with the number of hours slept, which was reported by each participant but not directly measured. Future research should consider including an objective measurement of these lifestyle variables as a source of improvement to the present investigation.

Additionally, conducting future prospective studies that validate the accuracy of the classification of the present CRT model in prognosis would help to confirm the present findings.

## 5. Conclusions

The CRT model found that hours of sleep, PA group, gender, age, education level, sedentary time, and diet are significant variables for classifying the diabetic population into those who suffer diabetes eye disease or do not.

The odds ratio and relative risks combined with the CRT model were used to identify the diabetes sufferers with a agreater risk of having their vision affected. Diabetes sufferers from the Inactives or Walking/Cycling PA groups had a greater risk of having their eyes affected by the disease than those with greater PA. Males from the Low, Moderate, and High PA groups had increased odds of suffering retinopathy than females from the same PA groups. Those from the Inactives or Walking/Cycling PA groups, and who slept fewer than 6 h or more than 9, had greater odds of this disease complication than those sleeping 6 to 8 h. Finally, diabetes sufferers aged over 62, who slept fewer than 6 h or more than 9, and who belonged to the PA groups Inactives or Walking/Cycling, had a greater risk of having their vision affected than those with the same PA and sleep habits, but who were younger.

## Figures and Tables

**Table 1:** Characterization and gender differences of the US diabetes sufferers aged 20 or more from the NHANES 2011–2020.

Variable							
Age	General Population (n = 2657)		Male (n = 1537)		Female (n = 1120)		*p*
Median (IQR)	63.00	(60)	64.00	(60)	61.00	(60)	<0.001
Mean (SD)	61.55	(12.82)	62.83	(12.05)	59.81	(13.62)	-
Diet	General Population (n = 2657)		Male (n = 1537)		Female (n = 1120)		*p* †
	n	%	n	%	n	%	
Poor	211	(7.9)	83	(5.4)	128 *	(11.4)	<0.001
Fair	737	(27.7)	407	(26.5)	330	(29.5)	
Good	1095	(41.2)	636	(41.4)	459	(41.0)	
Very Good	449	(16.9)	284	(18.5)	165 *	(14.7)	
Excellent	165	(6.2)	127	(8.3)	38 *	(3.4)	
Education level	General Population (n = 2657)		Male (n = 1537)		Female (n = 1120)		*p* †
	n	%	n	%	n	%	
Less than 9th grade	323	(12.2)	219	(14.3)	104 *	(9.3)	<0.001
9-11-12th grade with no diploma	393	(14.8)	208	(13.6)	185 *	(16.5)	
High school graduate/GED or equivalent	624	(23.5)	347	(22.6)	277	(24.7)	
College or AA degree	830	(31.3)	449	(29.3)	381 *	(34.0)	
College graduate or above	484	(18.2)	311	(20.3)	173 *	(15.4)	
High alcohol consumption	General Population (n = 2657)		Male (n = 1537)		Female (n = 1120)		*p* †
	n	%	n	%	n	%	
No	2110	(79.4)	1112	(72.3)	998	(89.1)	<0.001
Yes	547	(20.6)	425	(27.7)	122	(10.9)	
Sleep hours	General Population (n = 2657)		Male (n = 1537)		Female (n = 1120)		*p* †
	n	%	n	%	n	%	
Less than 5	152	(5.7)	79	(5.1)	73	(6.5)	0.455
5	231	(8.7)	129	(8.4)	102	(9.1)	
6	505	(19.0)	295	(19.2)	210	(18.8)	
7	642	(24.2)	392	(25.5)	250	(22.3)	
8	640	(24.1)	366	(23.8)	274	(24.5)	
9	296	(11.1)	167	(10.9)	129	(11.5)	
More than 10	191	(7.2)	109	(7.1)	82	(7.3)	
PA group	General Population (n = 2657)		Male (n = 1537)		Female (n = 1120)		*p* †
	n	%	n	%	n	%	
Inactives	980	(36.9)	504	(32.8)	476 *	(42.5)	<0.001
Walkers	183	(6.9)	111	(7.2)	72	(6.4)	
Low PA	538	(20.2)	309	(20.1)	229	(20.4)	
Moderate PA	477	(18.0)	283	(18.4)	194	(17.3)	
High PA	479	(18.0)	330	(21.5)	149 *	(13.3)	
Sedentary time (min)	General Population (n = 2657)		Male (n = 1537)		Female (n = 1120)		*p*
Median (IQR)	360.00	1320	360.00	1140	360.00	1320	0.362
Mean (SD)	388.79	213.74	393.63	217.09	382.14	208.97	-

*p* (*p*-value of the Mann–Whitney U test), *p* † (*p*-value of chi-square test). * (significant differences between proportions of men and women at 95% z-test for independent proportions).

**Table 2:** Importance of the independent variables, cross-validation, and accuracy of the CRT model.

	Absolute	Normalized Importance (%)
Sleep hours	0.002	100.0%
PA group	0.002	92.8%
Gender	0.002	76.2%
Age	0.001	46.4%
Education level	0.001	38.4%
Sedentary time	0.001	38.1%
Diet	<0.001	10.0%
Cross-validation	Estimation (SE)	
	0.206 (0.008)	
Model Accuracy	79.4%	

SE: standard error.

**Table 3:** Odds ratio and relative risk of having a negative perception of health, according to kissing frequency and relationship quality.

Branch Beneath Root Node		OR	95% CI	RR	95% CI	Rho	*p*
Inactives and Walking/Cycling	Low, Moderate, and High PA	1.48	1.22–1.78	1.36	1.17–1.58	0.079	<0.001 *
Branch beneath Node 1		OR	95% CI	RR	95% CI	rho	*p*
Male	Female	1.77	1.32–2.37	1.61	1.26–2.06	0.100	<0.001 *
Branch beneath Node 2		OR	95% CI	RR	95% CI	rho	*p*
Sleeps less than 5, 5, 9 or 10 h or more	Sleeps 6, 7 or 8 h	1.61	1.23–2.11	1.43	1.17–1.75	−0.101	0.001 *
Branch beneath Node 5		OR	95% CI	RR	95% CI	rho	*p*
Age > 62.5	Age ≤ 62.5	1.53	1.06–2.22	1.40	1.04–1.89	0.084	0.023 *

OR (odds ratio); CI (confidence interval); RR (relative risk); *p* (*p*-value); rho (Spearman’s correlation coefficient); *p* (*p*-value of the rho Spearman’s correlation coefficient). * *p*-value < 0.05.

**Table 4:** Binary logistic regression model with backward method for suffering diabetic eye disease.

Variable	B	SE	Wald	df	*p*	Exp (B)	95% CI	
							Lower	Upper
Gender	0.186	0.105	3.126	1	0.077	1.204	0.980	1.480
Age	0.007	0.004	2.944	1	0.086	1.007	0.999	1.015
Diet	-	-	2.896	4	0.575	-	-	-
Education Level	-	-	3.221	4	0.522	-	-	-
High alcohol consumption	0.063	0.121	0.275	1	0.600	1.065	0.841	1.350
Sleep Hours (7 h)	-	-	16.247	6	0.012	-	-	-
Sleep Hours (fewer than 5 h)	0.701	0.205	11.656	1	0.001 *	2.017	1.348	3.017
Sleep Hours (5 h)	0.211	0.191	1.225	1	0.268	1.235	0.850	1.796
Sleep Hours (6 h)	−0.042	0.157	0.070	1	0.791	0.959	0.705	1.305
Sleep Hours (8 h)	0.126	0.143	0.773	1	0.379	1.134	0.857	1.502
Sleep Hours (9 h)	0.082	0.179	0.212	1	0.645	1.086	0.764	1.543
Sleep Hours (10 h or more)	0.356	0.197	3.282	1	0.070	1.428	0.971	2.100
PA Group (Moderate PA)	-	-	12.666	4	0.013 *	-	-	-
PA Group (Inactives)	0.394	0.150	6.846	1	0.009 *	1.482	1.104	1.991
PA Group (Walking/Cycling)	0.668	0.211	10.009	1	0.002 *	1.950	1.289	2.949
PA Group (Low PA)	0.209	0.168	1.543	1	0.214	1.232	0.886	1.713
PA Group (High PA)	0.208	0.177	1.384	1	0.239	1.231	0.871	1.740
Sedentary time	0.000	0.000	2.299	1	0.129	1.000	1.000	1.001
CONSTANT	−1.117	0.370	9.133	1	0.003	0.327		

SE (standard error); *p* (*p*-value); * (*p*-value < 0.05).

## Data Availability

The data presented in this study are available on request from the corresponding author. The data are not publicly available due to privacy and ethical restrictions.
